# Willingness to Test for HIV and Initiate Post-exposure Prophylaxis Among Adolescent Girls and Young Women Seeking Emergency Contraceptive Pills in Pharmacies in Nairobi and Kisumu, Kenya: A Longitudinal Study

**DOI:** 10.1097/JNC.0000000000000626

**Published:** 2026-02-11

**Authors:** Nicholas Kipkurui, Gerald Owuor, Dorothy Oketch, Alloys K'Oloo, Scholastica Wanjiru, Kawango Agot, Alison C. Roxby

**Affiliations:** Nicholas Kipkurui, RN, PGD, is a Research Nurse, Impact Research Development Organization, Kisumu, Kenya.; Gerald Owuor, BA, is a Study Coordinator, Impact Research and Development Organization, Kisumu, Kenya.; Dorothy Oketch, MBChB, is a Research Officer, Afya Research Africa, Nairobi, Kenya.; Alloys K'Oloo, BSc Statistics, is a Data Officer, Kenya Medical Research Institute—Centre for Global Health Research, Kisumu, Kenya.; Scholastica Wanjiru, BPharm, is a Research Pharmacist, Impact Research and Development Organization, Kisumu, Kenya.; Kawango Agot, PhD, MPH, is a Principal Investigator, Impact Research and Development Organization, Kisumu, Kenya.; Alison C. Roxby, MD, Msc, is an Associate Professor, Departments of Medicine, Global Health, and Epidemiology, University of Washington, Seattle, Washington, USA.

**Keywords:** adolescent girls and young women, emergency contraceptives, HIV testing, pharmacy, post-exposure prophylaxis

## Abstract

Adolescent girls and young women (AGYW) often seek emergency contraceptive pills (ECP) within 72 hr of condomless sex, a critical period for HIV exposure prevention through testing and postexposure prophylaxis (PEP). Pharmacies, where most AGYW obtain ECP, provide an ideal setting for timely HIV prevention services. From May to October 2023, a longitudinal study was conducted among AGYW ages 15–24 years seeking ECP in five pharmacies in Nairobi and Kisumu. Eligible participants were not on preexposure prophylaxis and provided consent. Baseline and 10-day optional follow-up phone data assessed HIV testing history, risk perception, and willingness to test or start PEP. Of 297 screened, 200 enrolled (mean age = 22 years). Nearly all (97.5%) had previously tested for HIV; 79% were interested in pharmacy-based testing and 73% in PEP. By follow-up, 50.7% tested for HIV, and 10.5% initiated PEP. These findings suggest that pharmacies offer a promising avenue to link ECP users to timely HIV prevention service.

Despite significant advances in HIV prevention initiatives targeting priority populations, adolescent girls and young women (AGYW) continue to shoulder a disproportionate burden of new infections. Globally, AGYW ages 15–24 years account for 45% of the 1.3 million new cases of HIV in 2024, with 63% of these infections reported in sub-Saharan Africa (Joint United Nations Programme on HIV/AIDS [UNAIDS], 2024a). Furthermore, it is estimated that approximately 4,000 new HIV infections occur each week among AGYW ages 15–24 years (UNAIDS, 2024b). In Kenya, where the national HIV prevalence stands at 3.3%, females ages 15–49 years recorded a prevalence of 4.5% in 2024, twice the rate observed in males in the same age group at 2.2% (National Syndemic Disease Control Council, 2024).

Social determinants and individual-level risk factors driving HIV vulnerability among AGYW include gender inequality and poverty (Butts et al., 2017), sexual and gender-based violence (Bhattacharjee et al., 2020; Orindi et al., 2020), engagement in multiple concurrent short-term sexual relationships (Melesse et al., 2020), engaging in age-disparate transactional sexual relationships (Beauclair et al., 2018), alcohol use and low-risk perception (Price et al., 2018), and a limited awareness and uptake of HIV prevention interventions (Hill et al., 2020). Despite elevated risk of HIV acquisition and other sexually transmitted infections (STIs) associated with condomless sex, AGYW are often more concerned about preventing unwanted pregnancy due to its immediate social and economic consequences (Daniel et al., 2023). Consequently, many seek emergency contraceptive pills (ECP) for pregnancy prevention from pharmacies (Pintye et al., 2023). Studies have shown that pharmacies play a significant role as a primary provider of family planning options, including oral contraceptive pills, ECP, and condoms, especially among underserved populations (Aloo et al., 2023; Belachew et al., 2017; Corroon et al., 2016; Decker et al., 2022). Furthermore, observational studies have shown that private pharmacies provide a setting for differentiated delivery of HIV prevention and treatment services (Omollo et al., 2023) and are preferred by AGYW due to their convenience, privacy, and nonstigmatizing environment (Roche et al., 2021).

HIV postexposure prophylaxis (PEP) is evidence-based strategy for preventing HIV infection when taken immediately after condomless sex with a partner who is either living with HIV or whose HIV status is unknown. PEP is commonly administered following sexual assault, but it can also be used immediately after consensual sex encounters. However, PEP remain difficult to access, as initiation must occur within 72 hr of potential exposure. In Kenya, pharmacies have increasingly participated in delivering innovative HIV services, including HIV testing (McKeirnan et al., 2021) and preexposure prophylaxis (PrEP) provision (Bellman et al., 2022). AGYW frequently visit these pharmacies to purchase ECP (Gonsalves, Wyss, Gichangi, and Hilber, 2020), which are among the most commonly used family planning methods within this age group, and available for approximately 200 Kenyan Shillings (200 KES), equivalent to $2 . Because ECP are typically taken shortly after condomless sex, AGYW seeking ECP represent a potential target population for PEP provision within the critical 72-hr window. Pharmacies are often located within communities and offer more flexible hours than public health clinics, making them a convenient access point for sexual and reproductive health services. Linking AGYW seeking ECP to HIV prevention services at pharmacies may therefore provide a feasible approach to expanding access to proven HIV prevention interventions. In addition, the Kenyan government has made HIV self-testing available in its pharmacy sector at a cost of approximately KES 500 ($5; Ortblad, Mogere, et al., 2023, West et al., 2023). However, there is limited evidence on AGYWs' willingness to test and initiate PEP at pharmacies, particularly among those seeking ECP. Therefore, our study aimed to assess AGYWs' willingness to test for HIV and initiate PEP at the pharmacies, determine the proportion of AGYWs seeking ECP who expressed such willingness, and determine factors associated with willingness to test and initiate PEP, including risk perception, HIV testing history, and sociodemographic characteristics.

## Methods

### Study Design

We conducted a longitudinal observational study guided by the Health Belief Model, which posits that individuals' perceived susceptibility to a risk and their perceived benefits of an action influence health-seeking behaviors (Alyafei & Easton-Carr, 2024). Our study aimed to assess willingness to test for HIV and initiate PEP among AGYW seeking ECP at retail pharmacies in Nairobi and Kisumu. Our study population included AGYW ages 15–24 years who presented to participating pharmacies to obtain ECP. Using nonprobability consecutive sampling, trained pharmacy staff consecutively screened and enrolled eligible clients between May and October 2023. Inclusion criteria included being AGYW ages 15–24 years seeking ECP, not currently using PrEP, and able to provide informed written consent. Women older than 25 years or currently on PrEP were excluded. We set a target of sample size of 200 participants based on feasibility and comparability with prior pharmacy-based HIV prevention studies in Kenya (e.g., Pintye et al., 2023), which demonstrated adequate statistical precision for estimating willingness and uptake outcomes. A formal sample size calculation was not performed due to the exploratory nature of our study.

### Study Setting and Pharmacy Selection

We purposively selected Nairobi and Kisumu Counties to represent diverse urban contexts and HIV risk profiles among AGYW in Kenya. Nairobi represents a large metropolitan area with a high density of pharmacies and diverse socioeconomic populations, whereas Kisumu reflects a high HIV-burden setting in western Kenya, where AGYW experience elevated HIV incidence and frequently use pharmacy-based sexual and reproductive health services.

A list of registered pharmacies in both counties was obtained and categorized into small, medium, and large enterprises. *Small pharmacies* were defined as those operating with one or two pharmacy providers and serving fewer than 50 clients per day, and with or without a designated area for private consultations. *Medium pharmacies* employed three to five staff members, served approximately 50–150 clients daily, and maintained a designated area for private consultations or point-of-care testing. *Large pharmacies* were those integrated within chain outlets or larger retail establishments, staffed by more than five personnel, serving more than 150 clients per day, and offering multiple service points, including laboratory or diagnostic services. For our study, five community pharmacies across Nairobi and Kisumu counties were selected according to the following criteria: (1) registration and licensing by the Pharmacy and Poisons Board; (2) location in urban or periurban neighborhoods with high volumes of ECP sales and AGYW clientele, as documented in previous pharmacy-based sexual and reproductive health studies (Corroon et al., 2016; Pintye et al., 2023); (3) availability of private consultation area for participant engagement; and (4) willingness of pharmacy management to participate in our study. Pharmacies operating in low-demand areas or lacking privacy for client interactions were excluded.

### Pharmacy Staff Training

Each participating pharmacy received a comprehensive 2-day on-site training, scheduled in consultation with providers to minimize disruption to routine pharmacy operations. The training sessions covered our study protocol, participant screening and consenting procedures, research ethics, documentation, and data collection using REDCap. To accommodate the operational difference across the five pharmacies, the training was conducted over a 3-week period. Staff did not receive monetary compensation for participating in the training; however, appreciation tokens were provided after enrollment.

### Data Collection

Data were collected at two time points using electronic REDCap forms: baseline (at the pharmacy visit) and follow-up (through phone call 10 days post-enrollment). A trained pharmacy attendant conducted the initial screening at each participating pharmacy. All female clients who visited the pharmacy to purchase ECP during our study period were briefly approached in a private area and informed about our study. The attendants used a standardized checklist to confirm eligibility based on the following criteria: (1) ages 15–24 years, (2) seeking ECP, (3) not currently using PrEP, and (4) able and willing to provide informed consent. Clients who met the criteria and expressed interest were escorted to a private consultation area, where detailed explanation was provided and a written consent obtained. Screening outcomes—including total number of clients approached, eligible, ineligible, and enrolled—were documented daily in the REDCap system. Following eligibility screening and informed consent, participants completed a baseline survey at the pharmacy, either self-administered on a tablet or administered by a trained pharmacy attendant in a private consultation space. The baseline survey captured sociodemographic characteristics, ECP use, HIV testing history, HIV risk perception, and willingness to test for HIV or initiate PEP.

The pharmacy attendants provided participants with an informational sheet containing additional facts about HIV testing, PEP, and a toll-free counseling hotline managed by Liverpool Voluntary Counseling and Testing (LVCT, n.d.), a Kenyan-registered nongovernmental organization implementing innovative HIV prevention treatment and sexual reproductive health programs, as well as gender-based violence support services for vulnerable population (LCVT, n.d.). Participants were also invited to enroll in an optional follow-up phone survey conducted 10 days post-enrollment. Those who agreed provided a contact number and were later contacted by a trained research assistant (independent of the pharmacy attendant) to complete a brief phone-based questionnaire. The follow-up survey assessed whether participants had sought HIV testing or accessed PEP and explored facilitators and barriers to influencing service uptake.

In addition to client-level data, descriptive information about the participating pharmacies was collected, including location (urban or peri-urban), type of ownership, years in operation, number of pharmacy staff, availability of private consultation space, and proximity to the nearest public or private health facility. These data assess service delivery environment and evaluate the feasibility of pharmacies as potential access points for integrating HIV testing and PEP services.

### Data Analysis

The survey instrument was adapted from previously validated tools used in previous HIV prevention studies among AGYW in Kenya and comparable settings (Kamire et al., 2022). Internal consistency for the HIV knowledge and risk perception subscales was assessed using Cronbach alpha (α = 0.82), indicating good reliability. Composite indices for HIV risk perception and PEP knowledge were created by summing item responses, with higher scores reflecting greater perceived risk or knowledge. The data were imported directly from a REDCap electronic system hosted on a central server maintained by the University of Washington (Harris et al., 2019). Data cleaning involved logic and range checks, as well as frequency distributions, using Stata version 17 (Release 17, College Station, TX). Descriptive statistics were generated to summarize participant characteristics and key study variables. Bivariate analyses were used to assess associations between sociodemographic factors and interest in pharmacy-based HIV testing, and *p* values <.05 were considered statistically significant. Because different variables were measured at each time point, repeated-measures statistical analyses were not performed.

### Ethical Considerations

In Kenya, adolescent girls under 18 years commonly obtain ECP independently from community pharmacies, which are valued for their confidential and non-judgmental reproductive health services (Gonsalves, Wyss, Cresswell, et al., 2020). In recognition of this context, our study obtained a waiver of parental consent from the Institutional Review Board to avoid potential involuntary disclosure of sexual activity to parents or guardians. Participants ages 15–17 years provided written informed consent, and enrollment proceeded only after confirming their comprehension of study purpose and procedures. Pharmacy staff were trained to assess participant ability to make informed and independent decisions to ensure voluntary participation. This approach aligns with Kenya's national ethical guidelines for research involving minors and upholds the privacy and autonomy of adolescents seeking ECP and HIV prevention services.

Our study received ethical approval from the Kenya Medical Research Institute-Scientific and Ethics Review Unit (Ref. no Non-KEMRI-4574), and the National Commission for Science, Technology, and Innovation (Ref. no NACOSTI-P-23-23043). All participants provided written informed consent before enrollment. The consent form was available in both English and Kiswahili, and participants in Kisumu additionally had the option to use a Dholuo version. Our study instruments were originally developed in English and translated into Kiswahili and Dholuo following standard translation protocols. Two independent bilingual translators conducted forward translations, which were harmonized and subsequently back-translated into English to ensure conceptual equivalence. The research team reviewed and resolved discrepancies, and the finalized instruments were pre-tested with a small group of participants to assess clarity and accuracy.

## Results

### Pharmacy Characteristics

Of the five participating pharmacies, four were located in Kisumu and one in Nairobi. Pharmacy providers held diplomas in pharmacy, and each pharmacy had at least two providers. All pharmacies were located in urban informal settlements, with an average of operation of 3.5 years in operation and an average distance of 1.2 km away from the nearest public or private facility. All pharmacies offered point-of-care pregnancy testing and stocked oral HIV self-test kits, with some also providing blood-based HIV testing conducted on-site.

### Overview of Participants' Flow

**Figure 1.**
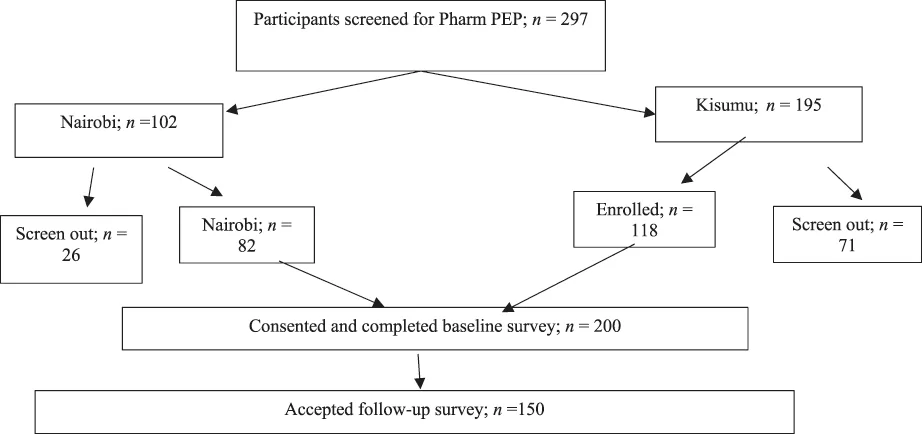
Flow diagram for participants.

Overall, 297 participants were screened and 200 participated in the survey between May and October 2023 (Figure 1).

### Participant Characteristics

Of the 297 participants screened, 97 were deemed ineligible: 54 were already using oral PrEP, and 45 were ages 25 years or older. Of those enrolled, the mean age was 22 years; regarding relationship status, 39% were dating, whereas 51% were single. In terms of education, 41.5% had completed secondary and 48.5% had attained postsecondary education. A majority (57%) of participants depended on their family for financial support, whereas 26% were self-employed. Most of the participants (97.5%) had previously undergone HIV testing before, with 52.3% testing more than 6 months prior. A majority, 68.4%, reported using self-test kits obtained at the pharmacy, 25% tested at public health facilities, and 6.6% at private facilities. HIV risk perception was relatively high, with 41.5% and 28.5% reporting high and moderate HIV risk, respectively. A total of 79% expressed interest in pharmacy-based HIV testing, with 37% preferring provider-administered testing, and 62.6% preferring self-testing. Despite the high self-perceived HIV risk, more than half (52.3%) had not tested within the previous 6 months. Furthermore, 76.9% expressed comfort in using an HIV self-test together with their sexual partners to know both partners' HIV status. Awareness of PEP was high (86.5%), and 73% expressed interest in pharmacy-initiated PEP; however, only 11.8% had ever used PEP in the past (Table 1).

**Table 1.** Characteristics of Adolescent Girls and Young Women Seeking ECP at Pharmacies

Characteristic	*N* (%)
County	
Nairobi	82 (41)
Kisumu	118 (59)
Age (years)	
15–19	38 (19)
20–24	162 (81)
Education level (completed/ongoing)	
Primary	20 (10.0)
Secondary	83 (41.5)
College/university	97 (48.5)
Relationship status	
Single	102 (51.0)
Dating	78 (39.0)
Married/in-union	17 (8.5)
No answer	3 (1.5)
Main source of income	
Family	114 (57.0)
Employed informal/formal sector	34 (17.0)
Self-employed	52 (26.0)
HIV risk perception	
Ever been tested for HIV before	
Yes	195 (97.5)
No	5 (2.5)
Last tested for HIV	
Within the last 3 months	58 (30.1)
4–6 months ago	34 (17.6)
More than 6 months	108 (52.3)
Where ever tested for HIV	
At a public facility/private clinic	112 (58.0)
At the pharmacy (self-test kit)	42 (21.8)
Used HIV self-test kit from elsewhere	32 (16.6)
Other	14 (7.2)
HIV risk perception	
High risk	83 (41.5)
Medium risk	57 (28.5)
Lower risk	46 (23.0)
No chance at all	11 (5.5)
No answer	3 (1.5)
Acceptability of pharmacy-based HIV testing and PEP	
Interest in pharmacy-based HIV testing	
Not at all interested	7 (3.5)
Somewhat interested	35 (17.5)
Interested	84 (42.0)
Very interested	74 (37.0)
How would you like to test at the pharmacy/(hypothetical)	
Pharmacy provider to test me	70 (37.0)
Use HIV self-test kit	117 (62.6)
No answer	13 (6.5)
Comfort with having HIV self-test with a sexual partner	
Very comfortable	62 (39.4)
Comfortable	59 (37.5)
Neutral	29 (18.5)
Uncomfortable	6 (3.8)
Very uncomfortable	17 (10.8)
No answer	27(13.5)
Awareness of PEP	
Yes	173 (86.5)
No	27 (13.5)
Ever used PEP	
Yes	20 (11.8)
No	150 (88.2)
No answer	30 (15)
Interest in initiating PEP at the pharmacy	
Very interested	86 (43.0)
Interested	60 (30.0)
Somewhat interested	42 (21.0)
Not at all interested	12 (6.0)
Client STI risk perception, use of emergency contraception, family planning	
Number of participants	*N* = 200
Characteristic	*N* (%)
Use of ECP in the last year	
This is my first time	102 (51.0)
Three times within a year	75 (37.5)
More than three times within a year	23 (11.5)
Contraceptive methods ever used	
None	53 (26.5)
Oral contraceptive pill (birth control pill)	67 (33.5)
Condoms used in general	53 (26.5)
3-month DMPA injection	17 (8.5)
Implants	10 (5.0)
STI risk perception	
High risk	85 (42.5)
Medium risk	46 (23.0)
Low risk	59 (29.5)
No chance at all	10 (5.0)
Ever had STI symptoms in the past 6 months	
Brownish vaginal discharge	11 (5.5)
Smelly vaginal discharge	20 (10.0)
Itchiness or vaginal discomfort	50 (25.0)
None of the symptoms	119 (59.5)
Where treated for STI symptoms (*N* = 81)	
At a private hospital/clinic	7 (8.6)
At public hospital	14 (17.3)
Bought drugs at the pharmacy	40 (49.4)

*Note. N* = 200. DMPA=depot-medroxyprogesterone acetate; ECP = emergency contraceptive pills; PEP = postexposure prophylaxis; STI = sexually transmitted infection.

### Client Sexually Transmitted Infection Risk Perception, Use of ECP, and Family Planning

A majority of AGYW (51.0%) were first-time users of ECP, whereas 33.5 and 8.5% reported ever using oral contraceptive pills and 3-month depot-medroxyprogesterone acetate injection, respectively. Overall, STI risk perception was high, with 42.5% and 23% reporting high and medium risk, respectively. STI symptoms were also reported, including vaginal discomfort (25%) and vaginal discharge (15.5%) in the past 6 months. Among those reporting STI symptoms, nearly half (49.4%) preferred seeking treatment at the pharmacy (Table 1).

### Factors Associated with Interest in Pharmacy-Based HIV Testing

Specific sociodemographic and behavioral factors were significantly associated with interest in accessing pharmacy-based HIV testing. Participants whose last HIV test was conducted more than 3 months ago were significantly more likely to express interest in pharmacy-based HIV testing (OR = 2.34; 95% CI [1.19–4.58]; *p* = .014) compared with those who had tested within the past 3 months. Similarly, participants who perceived themselves as being at medium or high risk of HIV infection were more likely to be interested in pharmacy-based testing Odds Ratio (OR) = 3.68; 95% CI [1.88–7.21]; *p* < .001) than those with low or no perceived risk. By contrast, those with postsecondary education (OR = 0.39; 95% CI [0.21–0.75]; *p* = .005), those who were married, in a union, or previously married (OR = 0.50; 95% CI [0.26–0.96]; *p* = .039), and those who were already aware of PEP (OR = 0.40; 95% CI [0.17–0.92]; *p* = .032) were less likely to express interest in pharmacy-based HIV testing compared with their respective reference groups (Table 2).

**Table 2.** Factors Associated With Interest in Pharmacy-Based HIV Testing

Variable	Category	*N* (%)	Crude OR (95% CI)	*p* value
County	Nairobi	82 (41.0)	Ref	—
	Kisumu	118 (59.0)	1.19 (0.83–1.70)	.358
Age (years)	15–19	38 (19.0)	Ref	—
	20–24	162 (81.0)	1.53 (0.72–3.27)	.268
Education level	Primary/secondary	103 (51.5)	Ref	—
	Post-secondary	96 (48.0)	0.39 (0.21–0.75)	.005*
Relationship status	Single	102 (51.0)	Ref	—
	Married/in union/divorced/widow (other)	95 (47.5)	0.50 (0.26–0.96)	.039*
Source of income	Family support	114 (57.0)	Ref	—
	Employed	86 (43.0)	0.68 (0.36–1.27)	.225
Last HIV test	≤3 months	58 (29.0)	Ref	—
	>3 months	135 (67.5)	2.34 (1.19–4.58)	.014*
HIV risk perception	No chance/low risk	57 (28.5)	Ref	—
	Medium/high risk	140 (70.0)	3.68 (1.88–7.21)	<.001*
PEP awareness	No	173 (86.5)	Ref	—
	Yes	27 (13.5)	0.40 (0.17–0.92)	.032*

*Note.* PEP = postexposure prophylaxis.

* Statistically significant.

### Follow-up Outcome of HIV Testing Postenrollment

Of the 150 AGYW successfully followed up, only half (50.7%) reported linking to HIV prevention services, with most (68.4%) preferring to access these services through pharmacies than public (25%) or private (6.6%) health facilities. Few participants reported receiving PEP services, with only 10.5% having tested for HIV and initiated PEP. Reasons cited for not seeking HIV testing included low perceived risk (20.2%), procrastination (20.2%), lack of time (18.9%), and fear of knowing one's HIV status (10.8%; Table 3).

**Table 3.** Follow-up Self-Reported Outcomes 10 Days Post Pharmacy Counseling

Characteristic	Overall *N* =150 (%)
County	
Nairobi	54 (36.0)
Kisumu	96 (64.0)
Linked for HIV testing services	
Yes	76 (50.7)
No	74 (49.3)
Where sought HIV services (*N* = 76)	
Bought an HIV self-test at the pharmacy	52 (68.4)
Public facility	19 (25)
Private facility	5 (6.6)
Service received	
HIV testing only	68 (89.5)
HIV testing and PEP initiation	8 (10.5)
Reason for not testing for HIV (*N* = 74)	
The facility was far from where I stay	5 (6.8)
I felt I was not at risk	15 (20.2)
I know my partner's HIV status	7 (9.5)
No answer/did not want to disclose	8 (10.8)
I fear knowing my HIV status	8 (10.8)
Did not have time to seek HIV testing	14 (18.9)
Still planning to go to HIV testing	15 (20.2)
Other	2 (2.7)

*Note.* PEP = postexposure prophylaxis.

## Discussion

This survey of AGYW seeking ECP at local pharmacies demonstrated that this population was interested in receiving HIV prevention services through pharmacies and would consider PEP initiation at the same time as seeking ECP. Our main findings indicated that the majority of sexually active AGYW perceived themselves to be at moderate or high risk of acquiring HIV, with many having last tested more than 6 months prior, and more than half were comfortable undertaking HIV self-testing with their sexual partners. Most were aware of PEP; however, very few had ever used these services.

Despite not conducting any outreach activities and relying solely on the distribution of an informational sheet by pharmacy staff, we observed that some AGYW sought HIV testing or initiated PEP within 10 days of receiving the informational sheet. These findings are based on self-reported outcomes and should be interpreted as associative rather than causative. The observed actions likely reflect increased awareness and motivation following receipt of the information, but further research using a controlled study design would be required to establish a causal linkage. Our study population reported high awareness of PEP, but relatively few participants initiated it. This could be attributed to the fact that the majority of participants opted for pharmacy-based HIV self-testing, the results of which are not currently accepted for initiated PEP, and due to limited availability of PEP products at the pharmacy. In addition, AGYW taking the survey reported reasons for not testing for HIV, including low perceived risk, procrastination, awareness of their sexual partner's HIV status, fear of learning their own HIV status, distance to HIV testing facilities, and time constraints. These findings highlight a persistent disparity in perceived HIV risk and actual uptake of prevention services among AGYW, contributing to the gap in the HIV prevention cascade, consistent with findings from to other studies (Price et al., 2018).

AGYW expressed high interest in pharmacy-based HIV testing and PEP services offered alongside emergency contraceptive services, with the majority preferring HIV self-testing over provider-assisted testing. This finding underscores the potential of the pharmacy as an accessible and youth-friendly service point for HIV prevention. Other pharmacy-based studies have also found that young people were satisfied with the quick, transactional interaction with pharmacy personnel and that a patient-centered-PEP service model is feasible and acceptable (Ortblad, Kwach, et al., 2023). In addition, regulatory, professional, health care service delivery, civil society, and research organizations stakeholders in Kenya recently endorsed the pharmacy sector as a key point for delivering HIV prevention services (Ortblad et al., 2020). In our study, a few AGYW purchased HIV self-testing kits after being informed about the availability of HIV testing services and receiving an informational sheet. Additionally, AGYW expressed willingness to undergo HIV self-testing with their sexual partners, aligning with findings similar to those observed in the peer PrEP model study (McGowan et al., 2024). This further highlights the potential role of the pharmacy sector in enhancing HIV service access among AGYW seeking emergency contraceptives. These findings are consistent with results from other studies that report a high level of fidelity in pharmacy-based HIV service delivery, preference for blood-based HIV testing, and the strong feasibility of integrating pharmacy-based PrEP services (Ortblad, Mogere, et al., 2023).

About half of AGYW seeking ECP were first-time users but had a history of family planning use, indicating that pharmacies are effectively reaching a population with unmet family planning needs. However, this also implies that AGYW were more concerned with pregnancy than contracting HIV and other STIs, a finding similar to other studies showing that adolescents were more concerned about unwanted pregnancy than STI acquisition following condomless sex (Bixby Center for Global Health, 2022; Daniel et al., 2023; Mason et al., 2025). In addition, ECP use has been linked to lower condoms use and reduced engagement in protective behaviors (Wasie et al., 2012). In our study, STI risk perception among this group was relatively high with more than half of our participants reporting perceiving being at a moderate to high risk. Although a majority reported no STI symptoms, about one quarter reported experiencing vaginal discharge, and nearly half reported experiencing vaginal discomfort. A majority preferred the pharmacy as their point of treatment. This finding demonstrates the active utilization of the pharmacy sector by AGYW for accessing family planning services and treatment of STI-related symptoms, consistent with findings from other studies (Corroon et al., 2016). Moreover, other studies in Africa have indicated that AGYW seeking ECP are at a heightened risk of STIs and are willing to participate in STI screening, yet often defer care from public facilities, opting instead for self-medication through pharmacy visits (Avuvika et al., 2017; Wang et al., 2023). Thus, the integration of family planning and HIV and STI prevention services within pharmacies is warranted for this high-risk group, and addressing barriers to youth-friendly sexual and reproductive services utilization remains essential.

## Limitations

Our study had a number of limitations. The surveys were conducted at pharmacies within informal settlements, and findings might not apply to larger pharmacies serving more formal or higher-income settlements. Furthermore, the majority of AGYW who participated in the survey had postsecondary education, meaning their perspectives may not be representative of AGYW with less formal education. Additionally, post-enrollment surveys were conducted remotely, and responses on linkage to care for HIV testing and PEP initiation relied on self-report and were therefore subject to recall or social desirability bias.

## Conclusion

Our findings demonstrate that pharmacies are reaching AGYW who are actively engaging in condomless sex and are interested in HIV prevention; thus, pharmacy encounters for ECP may be a viable and high-yield location to enhance access to PEP services. Pharmacy-based services have been shown to be a cornerstone of differentiated service delivery; by expanding outreach to AGYW seeking ECP, pharmacies could identify a population in need of services who are not currently accessing PEP. Pharmacies provide AGYW with a less intimidating, more flexible environment to access HIV services, particularly for those who may face time constraints or fear stigma in health facilities, and are convenient, low-barrier access points for at-risk populations, which could potentially improve prevention efforts. Given the interest from clients, pharmacies' ability to offer HIV testing to those seeking ECP and to offer immediate initiation of PEP should be explored. Training and sensitizing pharmacists in adolescent-friendly service delivery will be critical for successful implementation. Our study underscores the potential of pharmacies to enhance access to HIV testing and PEP initiation among at-risk AGYW by providing a discreet and convenient outreach points. Integrating HIV testing and PEP services within pharmacies is therefore key to addressing gaps in HIV prevention and referral systems for adolescents.

## Disclosures

The authors declare no conflicts of interest.

## Data Accessibility

Data will be provided upon request and with concurrence of the Kenya Medical Scientific and Ethics Review Unit.

## Author Contributions

All authors on this article meet the four criteria for authorship as identified by the International Committee of Medical Journal Editors (ICMJE); all authors have contributed to the conception and design of the study, drafted or have been involved in reviewing this manuscript, reviewed the final version of this manuscript before submission, and agree to be accountable for all aspects of the work. Specifically, using the CRediT taxonomy, the contributions of each author are as follows: Conceptualization & Methodology: N. Kipkurui, G. Owour, K. Agot, and A. C. Roxby; Formal Analysis: N Kipkurui and G. Owour; Funding acquisition: K. Agot and A. C. Roxby; Administration: D. Oketch and S. Wanjiru; Supervision: N. Kipkurui, D. Oketch, S. Wanjiru, K. Agot, and A. C. Roxby; Validation: A. K'Oloo; Writing—original draft: N. Kipkurui, G. Owour, D. Oketch, S. Wanjiru, A. K'Oloo, K. Agot, and A. C. Roxby; Writing/Revising—N. Kipkurui, G. Owour, D. Oketch, S. Wanjiru, A. K'Oloo, K. Agot, and A. C. Roxby.

**Key Considerations**

- Pharmacies where adolescent girls and young women (AGYW) seek emergency contraception can serve as timely access points for HIV prevention services in Kenya, including HIV testing and postexposure prophylaxis (PEP) initiation.
- Seeking emergency contraceptive pills (ECP) is a marker of condomless sex and signals an opportunity to address both pregnancy and HIV prevention.
- Training pharmacy providers to identify and support AGYW at risk can improve uptake of HIV prevention services.
- Linking ECP services with PEP may reduce missed opportunities for HIV prevention within the 72-hr window.
- Strengthening referral systems ensures continuity of care beyond the immediate pharmacy visit.
